# Publisher Correction: Global hotspots for the occurrence of compound events

**DOI:** 10.1038/s41467-020-20502-8

**Published:** 2020-12-18

**Authors:** Nina N. Ridder, Andy J. Pitman, Seth Westra, Anna Ukkola, Hong X. Do, Margot Bador, Annette L. Hirsch, Jason P. Evans, Alejandro Di Luca, Jakob Zscheischler

**Affiliations:** 1 Australian Research Council Centre of Excellence for Climate Extremes, University of New South Wales, Sydney, NSW Australia; 2 School of Civil, Environmental and Mining Engineering, University of Adelaide, Adelaide, SA Australia; 3 Australian Research Council Centre of Excellence for Climate Extremes, Australian National University, Canberra, ACT Australia; 4 School for Environment and Sustainability, University of Michigan, Ann Arbor, MI USA; 5 Faculty of Environment and Natural Resources, Nong Lam University, Ho Chi Minh City, Vietnam; 6 Oeschger Centre for Climate Change Research, University of Bern, Bern, Switzerland; 7 Climate and Environmental Physics, University of Bern, Bern, Switzerland

**Keywords:** Climate sciences, Natural hazards

Correction to: *Nature Communications* 10.1038/s41467-020-19639-3, published online 24 November 2020.

The original version of this Article contained an error in the spelling of the author Hong X. Do, which was incorrectly given as X. Do Hong. This has now been corrected in both the PDF and HTML versions of the Article.

