# Supplementary material for: Global hotspots for the occurrence of compound events
Source: Nat Commun. 2020 Nov 24;11:5956. doi: 10.1038/s41467-020-19639-3 (PMC7687898; doi:10.1038/s41467-020-19639-3)
Supplement: Supplementary file 1 — Supplementary Information [file 41467_2020_19639_MOESM1_ESM.pdf]

Supplementary Material - Global hotspots for the occurrence of compound events

Nina N. Ridder[1], Andy J. Pitman[1], Seth Westra[2], Anna Ukkola[3], Hong X. Do[4,5], Margot Bador[1], Annette L. Hirsch[1], Jason P. Evans[1], Alejandro Di Luca[1] and Jakob Zscheischler[6,7]

[1] Australian Research Council Centre of Excellence for Climate Extremes, University of New South Wales, Sydney, AUS

[2] School of Civil, Environmental and Mining Engineering, University of Adelaide, Adelaide, South Australia, Australia

[3] Australian Research Council Centre of Excellence for Climate Extremes, Australian National University, Canberra, AUS

[4] School for Environment and Sustainability, University of Michigan, Ann Arbor, Michigan, USA

[5] Faculty of Environment and Natural Resources, Nong Lam University, Ho Chi Minh City, Vietnam

[6] Oeschger Centre for Climate Change Research and Climate and Environmental Physics, University of Bern, Bern, Switzerland

[7] Climate and Environmental Physics, University of Bern, Bern, Switzerland

Corresponding author: Nina N. Ridder (n.ridder@unsw.edu.au)

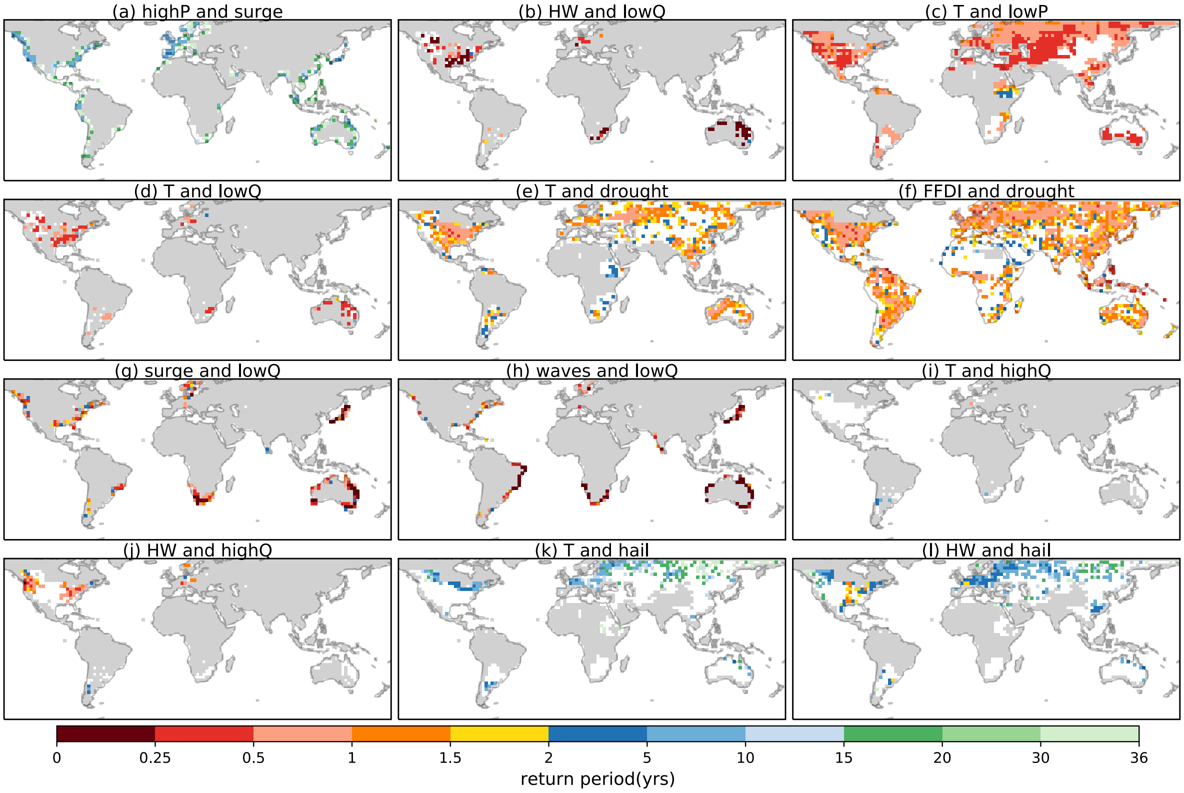

Supplementary Figure 1| Hotspots for the joint occurrence of the remaining hazard pairs including combinations of high precipitation (highP), high storm surge (surge), heatwaves (HW), low streamflow (lowQ), high temperatures (T), low precipitation (lowP), meteorological drought/low SPI (drought), high McArthur forest fire danger index values (FFDI), high wave heights (waves), high streamflow (highQ), and high probabilities of large hail (hail). Joint occurrences are given as return period of the actual probabilities derived from the data thereby allowing the direct comparison of different panels and to Fig. 1 and Supplementary Fig. 1. Only statistically significant values are shown ($p \leq 0.05$); statistically insignificant values are masked (white). Grey areas indicate regions without data coverage (Supplementary Fig. 9) or regions where percentile values of at least one hazard falls below the minimum required percentile value (Supplementary Table 2) or polar latitudes which are not taken into account in this study. Note that even though the data for T and highQ CEs in panel 1d are pointwise significant, the pair fails the field significance test (Supplementary Fig. 10). The pairing of high precipitation and meteorological drought (highP and drought) is omitted as it shows no statistically significant data points.

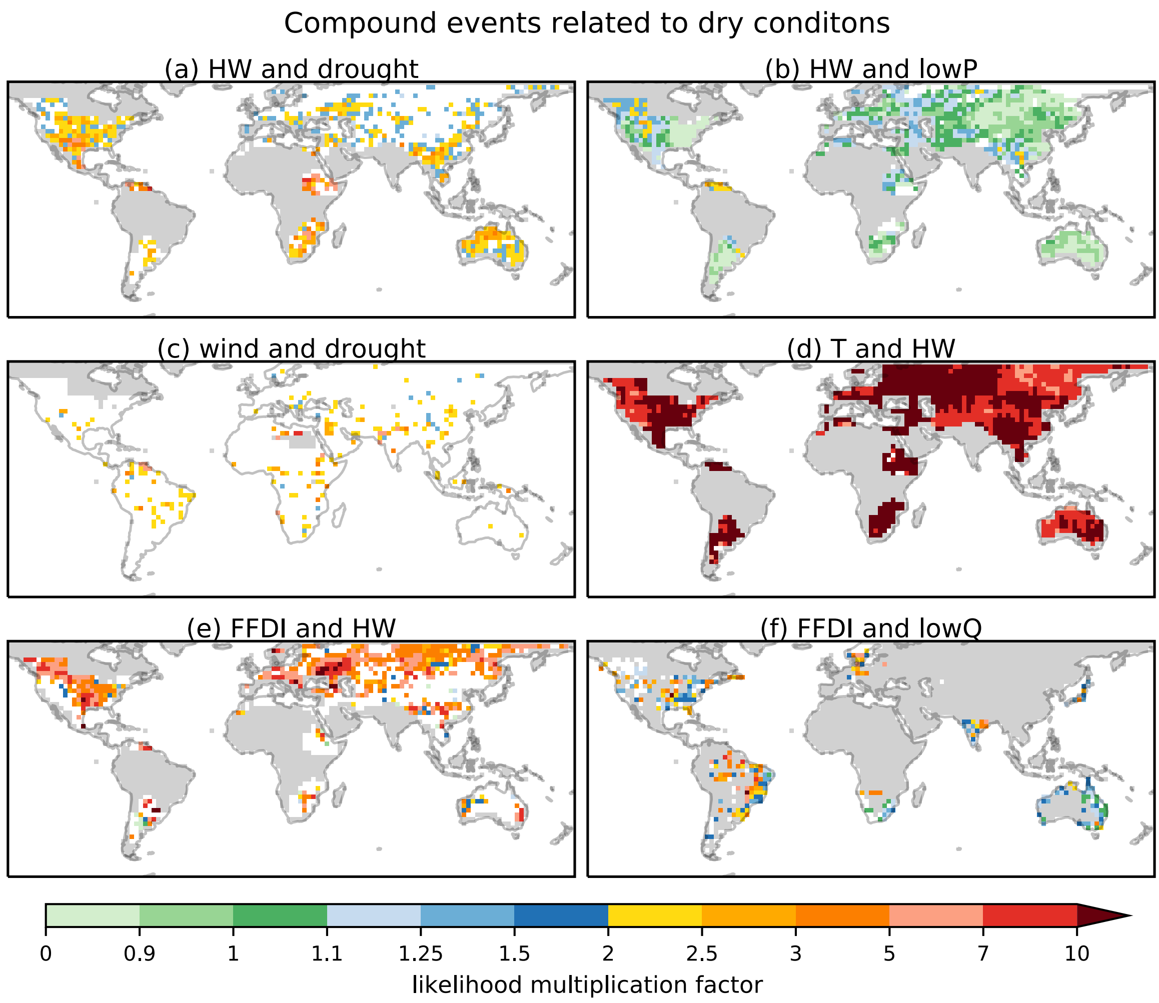

Supplementary Figure 2| Hotspots for the likelihood multiplication factor (LMF) of compound events related to dry conditions including meteorological drought (drought) and hydrological drought (lowQ) in combination with heatwaves (HW), extreme temperature (T), forest fire danger index (FFDI) and low streamflow (lowQ). Only statistically significant values are shown ($p \leq 0.05$); statistically insignificant values are masked (white). Grey areas indicate regions without data coverage (Supplementary Fig. 9), or regions where percentile values of at least one hazard falls below the minimum required percentile value (Supplementary Table 2) or polar latitudes which are not taken into account in this study.

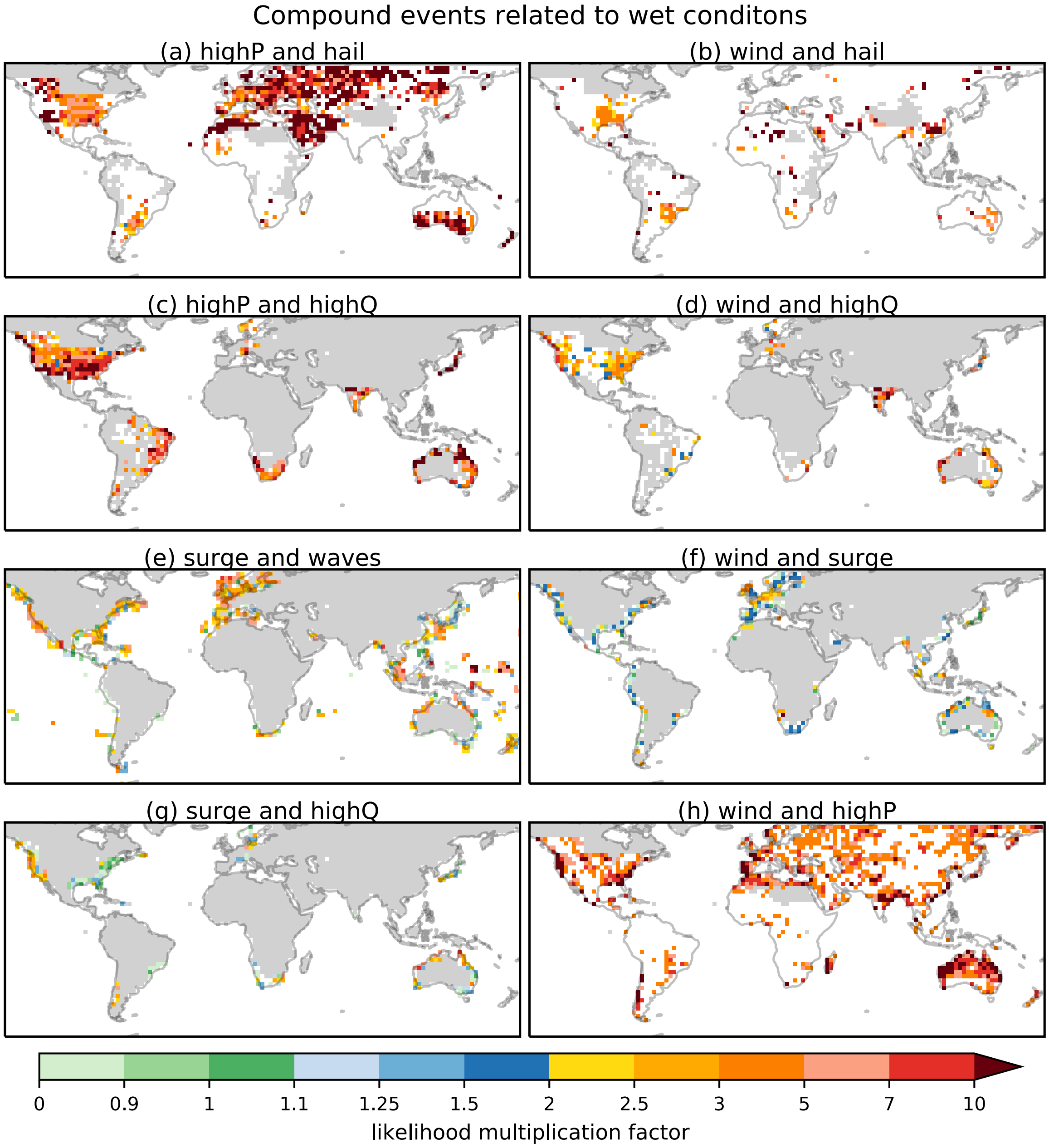

Supplementary Figure 3| Hotspots for the likelihood multiplication factor (LMF) of compound events related to wet conditions, combining high precipitation (highP), strong winds (wind), high probability of large hail (hail), high streamflow (highQ) and high storm surge (surge). Only statistically significant values are shown ($p \leq 0.05$); statistically insignificant values are masked (white). Grey areas indicate regions without data coverage (Supplementary Fig. 9), or regions where percentile values of at least one hazard falls below the minimum required percentile value (Supplementary Table 2) or polar latitudes which are not taken into account in this study.

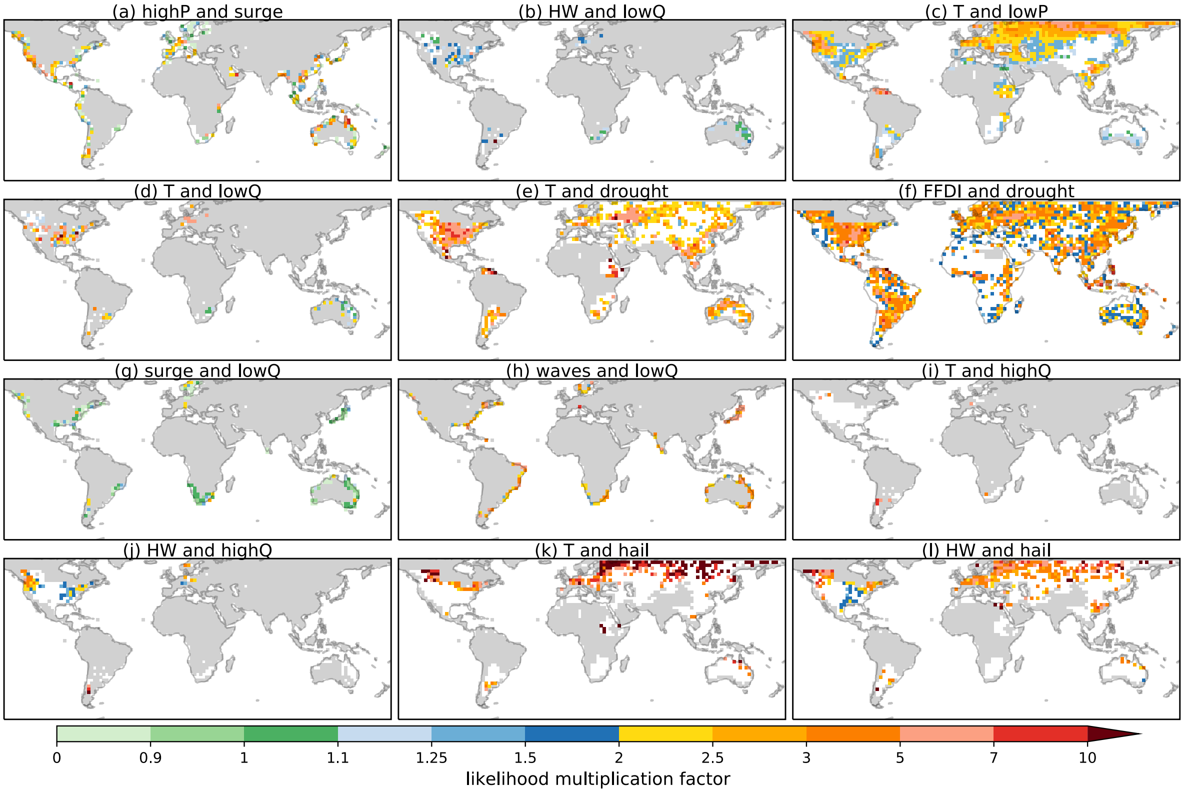

Supplementary Figure 4| Hotspots for the likelihood multiplication factor (LMF) of the remaining hazard pairs including combinations of high precipitation (highP), high storm surge (surge), heatwaves (HW), low streamflow (lowQ), high temperatures (T), low precipitation (lowP), meteorological drought/low SPI (drought), high McArthur forest fire danger index values (FFDI), high wave heights (waves), high streamflow (highQ), and high probabilities of large hail (hail). Only statistically significant values are shown ($p \leq 0.05$); statistically insignificant values are masked (white). Grey areas indicate regions without data coverage (Supplementary Fig. 9), or regions where percentile values of at least one hazard falls below the minimum required percentile value (Supplementary Table 2) or polar latitudes which are not taken into account in this study. Note that even though the data for T and highQ CEs in panel 1d are pointwise significant, the pair fails the field significance test (Supplementary Fig. 10). The pairing of high precipitation and meteorological drought (highP and drought) is omitted as it shows no statistically significant data points.

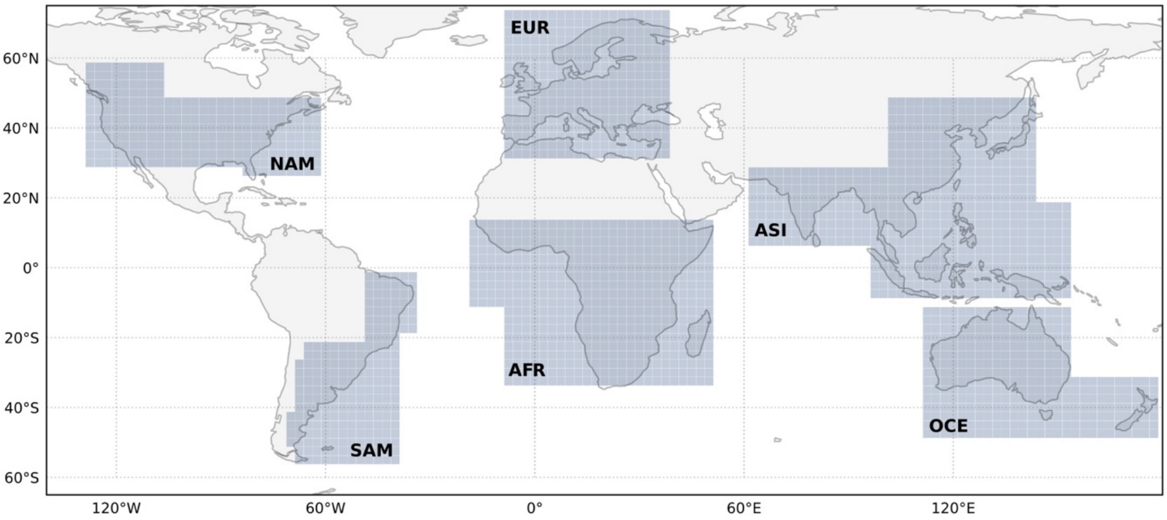

Supplementary Figure 5| Areas assessed in more detail in this study. Naming: NAM – North America; SAM – South America; EUR – Europe; AFR –Africa; ASI – Asia; OCE – Oceania.

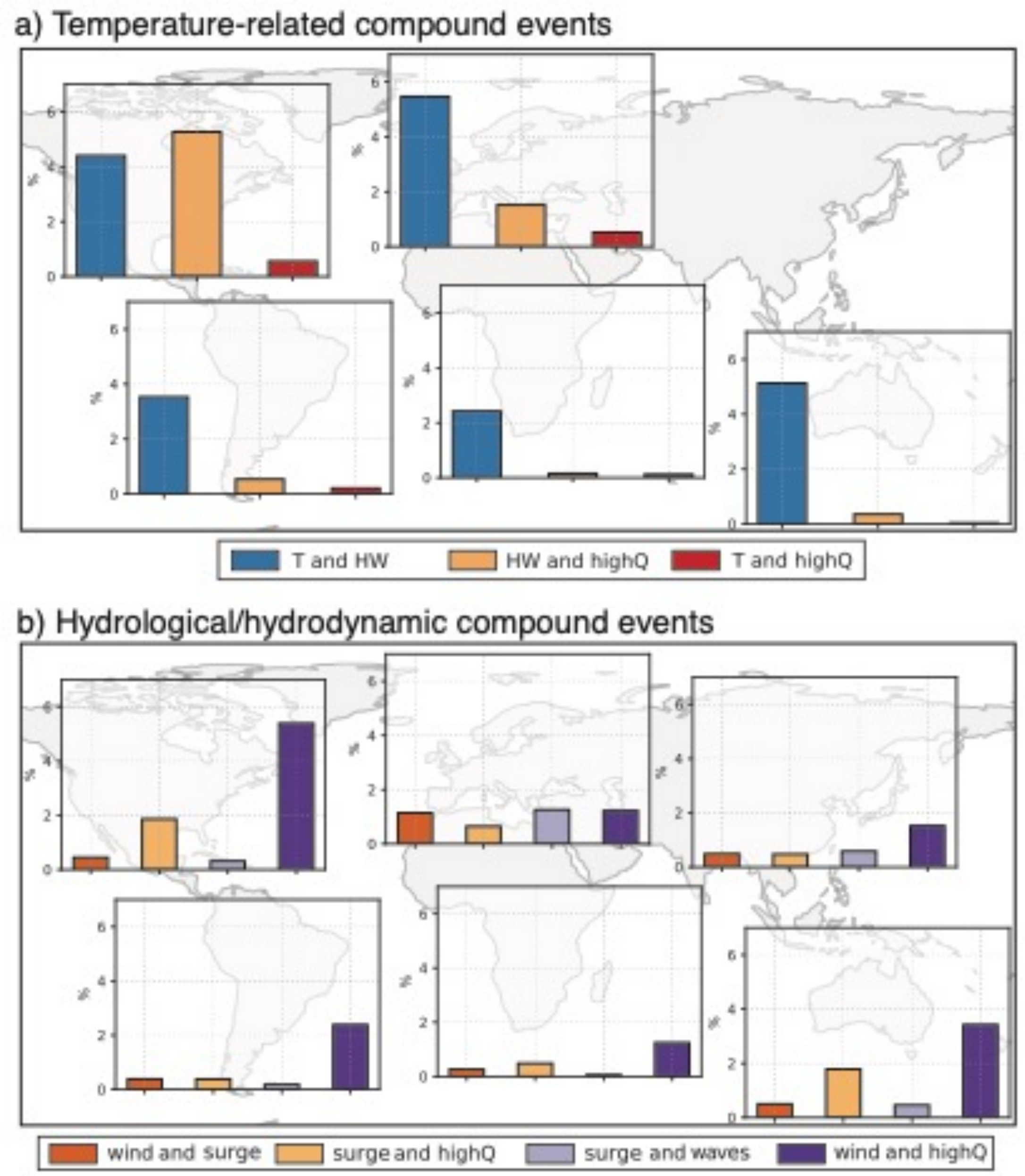

Supplementary Figure 6| Relative importance of hazard pair per region. Percentages are weighted by number of grid cells in the region taking into consideration the different data coverage of different hazard pairs (Supplementary Fig. 9). Comparable with Fig. 3 but for (a) temperature-related and (b) hydrologic/hydrodynamic compound events. Hazards included are high temperature (T), heatwaves (HW), high streamflow (highQ), strong winds (wind), high storm surge (surge), and high wave heights (waves). Note the different range of the y-axes between (a), (b), and Fig. 3.

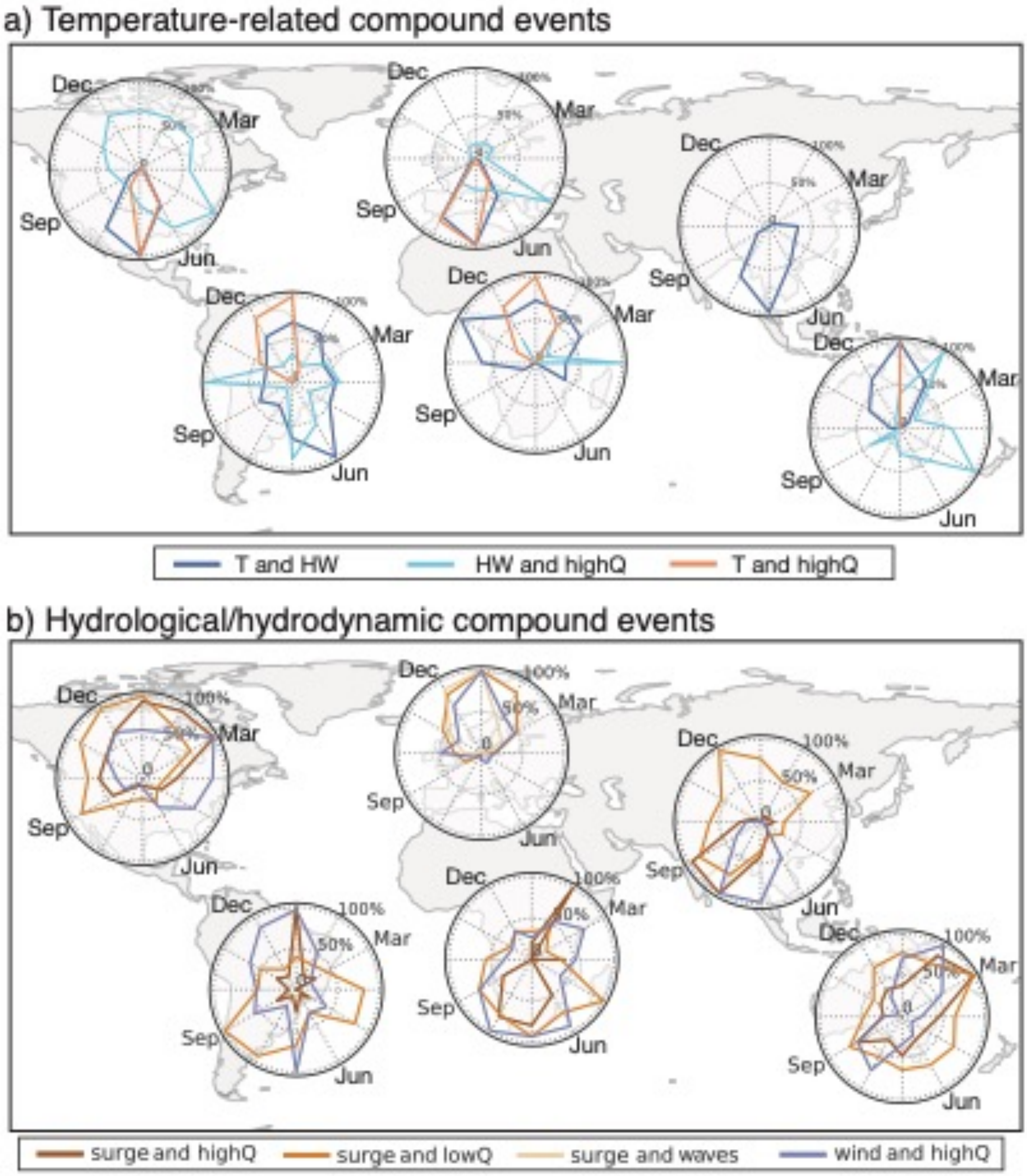

Supplementary Figure 7| Seasonality of the most dominant CE types per region. Values are relative to the total number of events in the month with the most occurrences comparable to Fig. 4 but for (a) temperature-related and (b) hydrologic/hydrodynamic compound events. The hazard combinations include high temperature (T), heatwaves (HW), low streamflow (lowQ), high streamflow (highQ), high storm surge (surge), high wave heights (waves), and strong winds (wind).

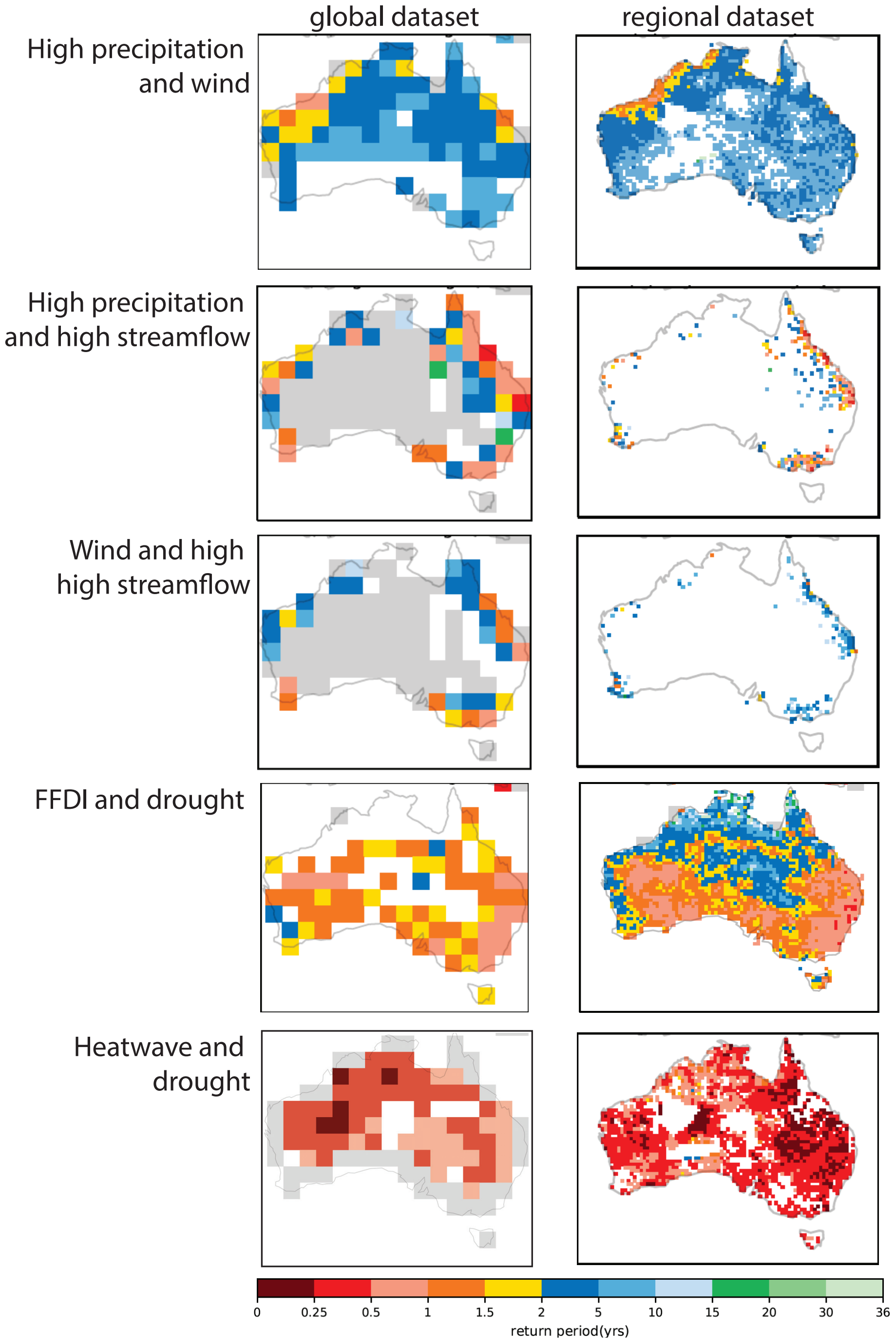

Supplementary Figure 8| Comparison between CE hotspots in Australia derived from global datasets (left; see Supplementary Table 2 for details) and regional high-resolution datasets (right). Shown are the most important hazard combinations for this region. Regional high-resolution data for precipitation, drought (SPI) and heatwaves (EHF) were taken from the Australian Water Availability Project (AWAP)[1]. High-resolution 10m winds were derived from ECMWF's ERA5's zonal and meridional 10m wind components. High-resolution FFDI data were taken from the Copernicus Emergency Management Service for the European Forest Fire Information System (EFFIS)[2]. Streamflow data were derived from a high-resolution gridded version of the global dataset[3, 4].

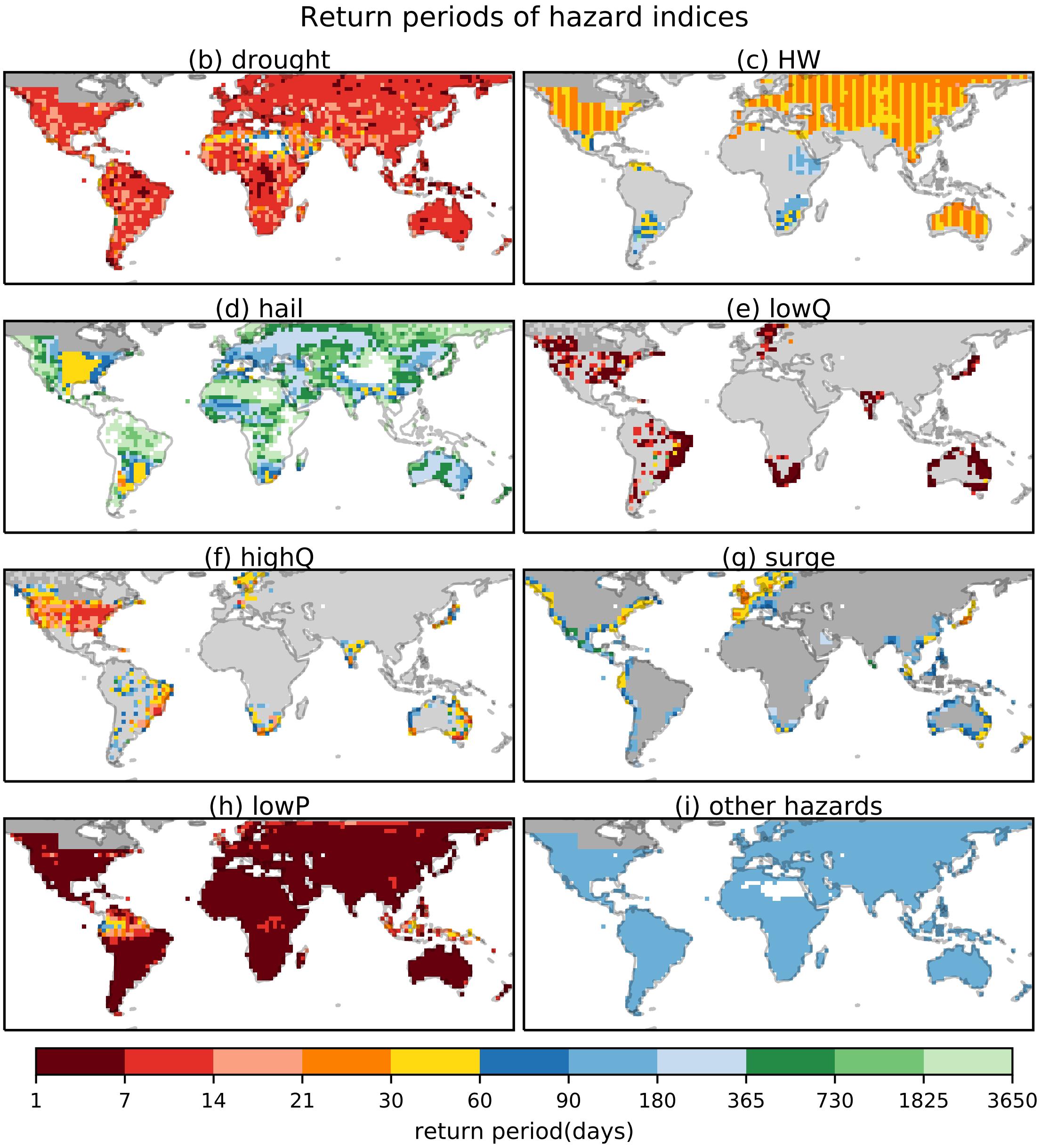

Supplementary Figure 9| Return periods for the different univariate hazards (a) low SPI (drought), (b) heatwaves (HW), (c) high probability of large hail (hail), (d) low streamflow (lowQ), (e) high streamflow (highQ), (f) low precipitation (lowP), and (g) all other hazards. Grey areas indicate regions without data coverage (Supplementary Fig. 9) or polar latitudes which are not taken into account in this study. White areas indicate regions where percentile values of the hazard fall below the minimum required percentile value (Supplementary Table 2).

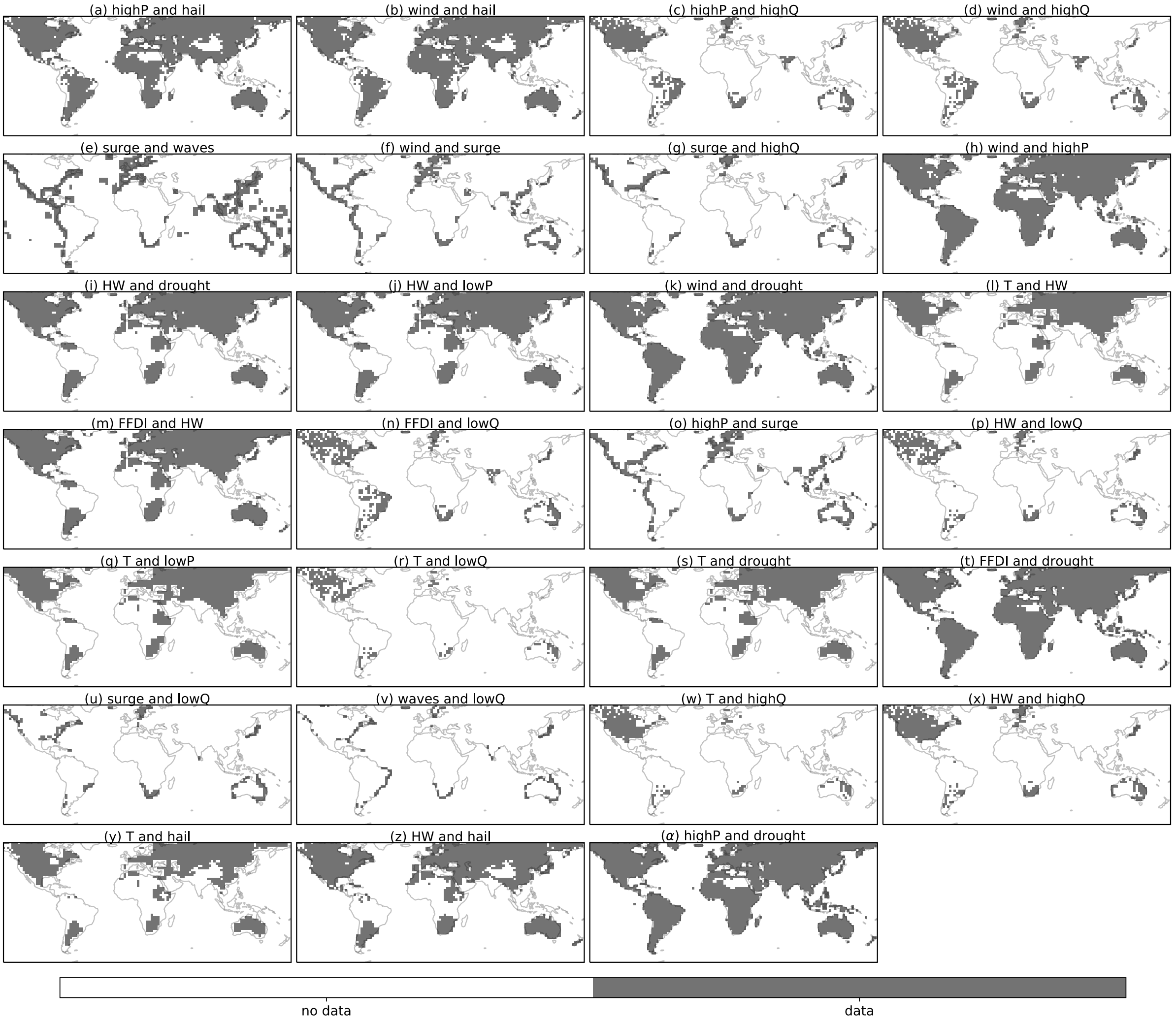

Supplementary Figure 10| Data coverage per hazard pair for CEs related to dry conditions consisting of combinations of heatwaves (HW), low SPI (drought), low precipitation (lowP), strong winds (wind), high temperatures (T), high McArthur forest fire index values (FFDI), low streamflow (lowQ), high precipitation (highP), high probability of large hail (hail), strong winds (wind), high streamflow (highQ), high storm surge (surge), extreme wave height (waves), and high probability of large hail (hail).

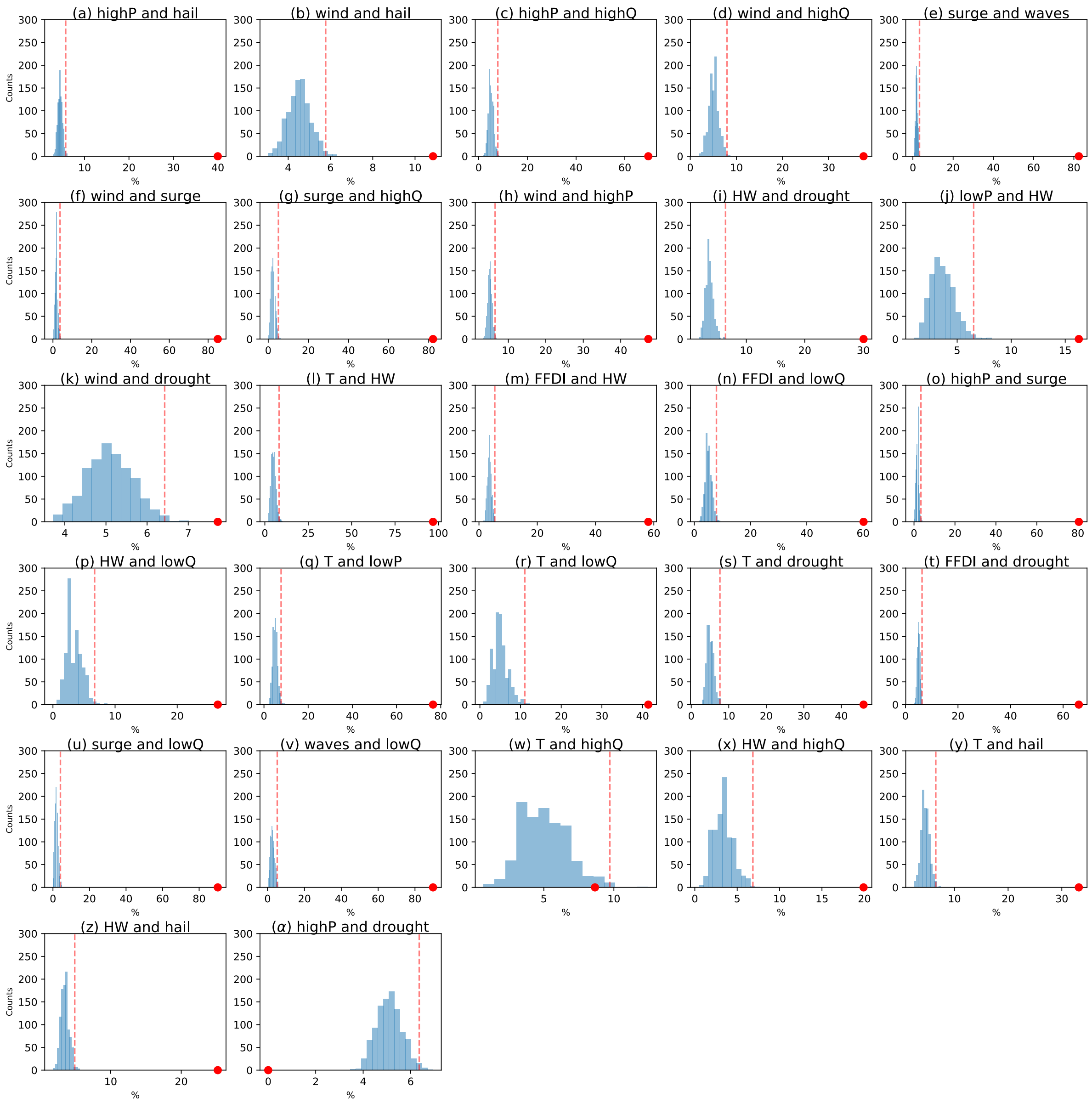

Supplementary Figure 11| Histograms of the distribution of statistically significant grid cells from 1000 bootstrap realizations of the joint exceedance for hazard pairs related to dry conditions. The x-axis is scaled by the total number of grid cells covered by a hazard pair (Supplementary Fig. 9). The red dot marks the number of statistically significant cells of the original joint exceedance count compared to the 1000 bootstrapped realizations. Results are considered field significant if this value is right of the dashed red line that indicates the 97.5% threshold in the resampled realisations set for field significance. The hazard pairs shown consist of combination of high precipitation (highP), high storm surge (surge), heatwaves (HW), low streamflow (lowQ), high temperatures (T), low precipitation (lowP), low SPI (drought), high McArthur forest fire danger index values (FFDI), high wave heights (waves), and high probability of large hail (hail). Note the different x-axes between the subplots.

Hazard pair/CE type	Abbreviation	Possible impact/consequences
Heatwave & drought	HW-drought	Crop failure; increased mortality; water shortages
Heatwave & low precipitation	HW-lowP	Crop failure; increased mortality; water shortages
High temperature & heatwave	T-HW	Increased mortality
Fire weather & heatwaves*	FFDI-HW	Increased wildfire risk; increased mortality; poor air quality; property damage
Fire weather & drought*	FFDI-drought	Increased wildfire risk; increased mortality; water shortages preventing firefighting; poor air quality; property damage
Fire weather & low streamflow*	FFDI-lowQ	increased mortality; water shortages; property damage; water shortages preventing fire fighting
High precipitation & large hail*	highP-hail	Property damage; crop failure; (flash) flooding
Wind & large hail*	wind-hail	Property damage
High precipitation & high streamflow	highP-highQ	Flooding; loss of life; property damage; crop failure
Wind & high streamflow*	wind-highQ	Flooding; loss of life; property damage
Surge & waves*	surge-waves	Coastal erosion; flooding; inland saltwater inundation leading to losses in agriculture
High precipitation & surge*	highP-surge	Coastal flooding
Wind & surge*	wind-surge	Storm damage and coastal flooding; coastal erosion
Surge & high streamflow*	surge-highQ	Flooding; coastal erosion
Wind & high precipitation*	wind-highP	Wind-driven rain and associated damages
High temperature & low precipitation	T-lowP	Crop failure; increased mortality; water shortages
High temperature & drought	T-drought	Crop failure; increased mortality; water shortages
High temperature & low streamflow	T-lowQ	Crop failure; water shortages
High temperatures & high streamflow	T-highQ	Spread of water-borne diseases
Heatwave & low streamflow	HW-lowQ	Crop failure; increased mortality; water shortages

High temperature & large hail*	T-hail	Property damage; increased mortality; crop failure
Surge & low streamflow*	surge-lowQ	Inland saltwater inundation
Heatwave & large hail*	HW-hail	Property damage; increased mortality; crop failure
Waves & low streamflow*	waves-lowQ	Inland saltwater inundation
High precipitation & drought	highP-drought	Flash flooding; crop failure
Heatwave & high streamflow	HW-highQ	Property damage; loss of life; Spread of water-borne diseases
Wind & drought*	wind-drought	Property damage; crop failure; Poor air quality

Supplementary Table 1| Hazard pairs considered in this study and examples for their potential impacts. Hazard pairs containing at least one hazard derived from reanalysis are marked with (*).

**Hazard**	**Name**	**Threshold**	**Cut off values**	**Data Source**
maximum temperature	T		< -1˚C	HadGHCND[5]
high streamflow	highQ		< 0 m^3/s	GSIM [3, 4]
high precipitation	highP		< 1 mm	REGEN [6]
wind speed*	wind	≥ 99th percentile	< 0.5 m/s	ERA-Interim [7]
storm surge*	surge		< 0.001 m	GTSM [8]
wave height*	waves		<0	CAWCR [9, 10]
McArthur Forest Fire Index*	FFDI		< 0	Derived from ERA-Interim data[11]
low precipitation	lowP	≤ 1st percentile	> 0 mm	REGEN
low streamflow	lowQ	≤ 10th percentile	< 0.01 m^3/s	GSIM
Standardised Precipitation Index (SPI)	drought	≤ -1.3	NA	REGEN
Excess Heat Factor (EHF)	HW	> 0	NA	HadGHCND
Probability for large hail*	hail	≥ 0.5	NA	[12]

Supplementary Table 2| Hazards taken into account in this study and their thresholds for the identification of compound events, the lowest allowed value of the chosen percentile and source of dataset. Hazards derived either directly (wind speed) or indirectly (e.g. surge height) from reanalysis products are marked with (*).

Supplementary References

1. Raupach M., Briggs P., Haverd V., King E., Paget M. & Trudinger C. Australian water availability project. *Canberra: CSIRO Marine and Atmospheric Research* (2012).

2. Vitolo C*., et al.* ERA5-based global meteorological wildfire danger maps. *Scientific data* **7**, 1-11 (2020).

3. Do H.X., Gudmundsson L., Leonard M. & Westra S. The Global Streamflow Indices and Metadata Archive (GSIM)-Part 1: The production of a daily streamflow archive and metadata. *Earth System Science Data* **10**, 765-785 (2018).

4. Gudmundsson L., Do H.X., Leonard M. & Westra S. The Global Streamflow Indices and Metadata Archive (GSIM)-Part 2: Quality control, time-series indices and homogeneity assessment. *Earth System Science Data* **10**, 787-804 (2018).

5. Caesar J., Alexander L. & Vose R. Large-scale changes in observed daily maximum and minimum temperatures: Creation and analysis of a new gridded data set. *Journal of Geophysical Research: Atmospheres* **111**, D05101 (2006).

6. Contractor S*., et al.* Rainfall estimates on a gridded network (REGEN) – a global land-based gridded dataset of daily precipitation from 1950 to 2016. *Hydrology and Earth System Sciences* **24**, 919-943 (2020).

7. Berrisford P*., et al.* The ERA-Interim Archive. In: *ERA report series*) (2011).

8. Muis S., Verlaan M., Winsemius H.C., Aerts J.C.J.H. & Ward P.J. A global reanalysis of storm surge and extreme sea levels (1979-2014). *Nature Communications* **7**, 1-11 (2016).

9. Durrant T., Hemer M., Trenham C. & Greenslade D. CAWCR Wave Hindcast 1979–2010. *Data Collection* (2013).

10. Durrant T., Hemer M., Smith G., Trenham C. & Greenslade D. CAWCR Wave Hindcast - Aggregated Collection. v1. *Service Collection* **1** (2019).

11. Vitolo C., Di Giuseppe F., Krzeminski B. & San-Miguel-Ayanz J. Data descriptor: A 1980–2018 global fire danger re-analysis dataset for the Canadian fire weather indices. *Scientific Data* **6**, 1-10 (2019).

12. Prein A. & Holland G. Daily gridded hail risk estimates on a global scale (1979 to 2015), link to netCDF files.). PANGAEA (2018).
